# Supplementary material for: Impact of joint commission international accreditation on occupational health and patient safety: A systematic review
Source: PLoS One. 2025 Jun 17;20(6):e0325894. doi: 10.1371/journal.pone.0325894 (PMC12173381; doi:10.1371/journal.pone.0325894)
Supplement: S2 File — (PDF) [file pone.0325894.s002.pdf]

Appendix 1

Study Quality Assessment Tools

In 2013, NHLBI developed a set of tailored quality assessment tools to assist reviewers in focusing on concepts that are key to a study's internal validity. The tools were specific to certain study designs and tested for potential flaws in study methods or implementation. Experts used the tools during the systematic evidence review process to update existing clinical guidelines, such as those on cholesterol, blood pressure, and obesity. Their findings are outlined in the following reports:

- Assessing Cardiovascular Risk: Systematic Evidence Review from the Risk Assessment Work Group
- Management of Blood Cholesterol in Adults: Systematic Evidence Review from the Cholesterol Expert Panel
- Management of Blood Pressure in Adults: Systematic Evidence Review from the Blood Pressure Expert Panel
- Managing Overweight and Obesity in Adults: Systematic Evidence Review from the Obesity Expert Panel

While these tools have not been independently published and would not be considered standardized, they may be useful to the research community. These reports describe how experts used the tools for the project. Researchers may want to use the tools for their own projects; however, they would need to determine their own parameters for making judgements. Details about the design and application of the tools are included in Appendix A of the reports.

Quality Assessment of Controlled Intervention Studies

- Study Quality Assessment Tools

Quality Assessment of Systematic Reviews and Meta-Analyses

- Study Quality Assessment Tools

Quality Assessment Tool for Observational Cohort and Cross-Sectional Studies

- Study Quality Assessment Tools

Quality Assessment of Case-Control Studies

- Study Quality Assessment Tools

Quality Assessment Tool for Before-After (Pre-Post) Studies With No Control Group

- Study Quality Assessment Tools

**Criteria**	**Yes**	**No**	**Other (CD, NR, NA)***
1. Was the study question or objective clearly stated?			
2. Were eligibility/selection criteria for the study population prespecified and clearly described?			
3. Were the participants in the study representative of those who would be eligible for the test/service/intervention in the general or clinical population of interest?			
4. Were all eligible participants that met the prespecified entry criteria enrolled?			
5. Was the sample size sufficiently large to provide confidence in the findings?			
6. Was the test/service/intervention clearly described and delivered consistently across the study population?			
7. Were the outcome measures prespecified, clearly defined, valid, reliable, and assessed consistently across all study participants?			
8. Were the people assessing the outcomes blinded to the participants' exposures/interventions?			
9. Was the loss to follow-up after baseline 20% or less? Were those lost to follow-up accounted for in the analysis?			
10. Did the statistical methods examine changes in outcome measures from before to after the intervention? Were statistical tests done that provided p values for the pre-to-post changes?			
11. Were outcome measures of interest taken multiple times before the intervention and multiple times after the intervention (i.e., did they use an interrupted time-series design)?			
12. If the intervention was conducted at a group level (e.g., a whole hospital, a community, etc.) did the statistical analysis take into account the use of individual-level data to determine effects at the group level?			

**Quality Rating (Good, Fair, or Poor) (see guidance)**
Rater #1 Initials:
Rater #2 Initials:
Additional Comments (If POOR, please state why):

*CD, cannot determine; NA, not applicable; NR, not reported

Guidance for Assessing the Quality of Before-After (Pre-Post) Studies With No Control Group

The guidance document below is organized by question number from the tool for quality assessment of controlled intervention studies.

Question 1. Study question

Did the authors describe their goal in conducting this research? Is it easy to understand what they were looking to find? This issue is important for any scientific paper of any type. Higher quality scientific research explicitly defines a research question.

Question 2. Eligibility criteria and study population

Did the authors describe the eligibility criteria applied to the individuals from whom the study participants were selected or recruited? In other words, if the investigators were to conduct this study again, would they know whom to recruit, from where, and from what time period?

Here is a sample description of a study population: men over age 40 with type 2 diabetes, who began seeking medical care at Phoenix Good Samaritan Hospital, between January 1, 2005 and December 31, 2007. The population is clearly described as: (1) who (men over age 40 with type 2 diabetes); (2) where (Phoenix Good Samaritan Hospital); and (3) when (between January 1, 2005 and December 31, 2007). Another sample description is women who were in the nursing profession, who were ages 34 to 59 in 1995, had no known CHD, stroke, cancer, hypercholesterolemia, or diabetes, and were recruited from the 11 most populous States, with contact information obtained from State nursing boards.

To assess this question, reviewers examined prior papers on study methods (listed in reference list) when necessary.

Question 3. Study participants representative of clinical populations of interest

The participants in the study should be generally representative of the population in which the intervention will be broadly applied. Studies on small demographic subgroups may raise concerns about how the intervention will affect broader populations of interest. For example, interventions that focus on very young or very old individuals may affect middle-aged adults differently. Similarly, researchers may not be able to extrapolate study results from patients with severe chronic diseases to healthy populations.

Question 4. All eligible participants enrolled

To further explore this question, reviewers may need to ask: Did the investigators develop the I/E criteria prior to recruiting or selecting study participants? Were the same underlying I/E criteria used for all research participants? Were all subjects who met the I/E criteria enrolled in the study?

Question 5. Sample size

Did the authors present their reasons for selecting or recruiting the number of individuals included or analyzed? Did they note or discuss the statistical power of the study? This question addresses whether there was a sufficient sample size to detect an association, if one did exist.

An article's methods section may provide information on the sample size needed to detect a hypothesized difference in outcomes and a discussion on statistical power (such as, the study had 85 percent power to detect a 20 percent increase in the rate of an outcome of interest, with a 2-sided alpha of 0.05). Sometimes estimates of variance and/or estimates of effect size are given, instead of sample size calculations. In any case, if the reviewers determined that the power was sufficient to detect the effects of interest, then they would answer "yes" to Question 5.

Question 6. Intervention clearly described

Another pertinent question regarding interventions is: Was the intervention clearly defined in detail in the study? Did the authors indicate that the intervention was consistently applied to the subjects? Did the research participants have a high level of adherence to the requirements of the intervention? For example, if the investigators assigned a group to 10 mg/day of Drug A, did most participants in this group take the specific dosage of Drug A? Or did a large percentage of participants end up not taking the specific dose of Drug A indicated in the study protocol?

Reviewers ascertained that changes in study outcomes could be attributed to study interventions. If participants received interventions that were not part of the study protocol and could affect the outcomes being assessed, the results could be biased.

Question 7. Outcome measures clearly described, valid, and reliable

Were the outcomes defined in detail? Were the tools or methods for measuring outcomes accurate and reliable–for example, have they been validated or are they objective? This question is important because the answer influences confidence in the validity of study results.

An example of an outcome measure that is objective, accurate, and reliable is death–the outcome measured with more accuracy than any other. But even with a measure as objective as death, differences can exist in the accuracy and reliability of how investigators assessed death. For example, did they base it on an autopsy report, death certificate, death registry, or report from a family member? Another example of a valid study is one whose objective is to determine if dietary fat intake affects blood cholesterol level (cholesterol level being the outcome) and in which the cholesterol level is measured from fasting blood samples that are all sent to the same laboratory. These examples would get a "yes."

An example of a "no" would be self-report by subjects that they had a heart attack, or self-report of how much they weight (if body weight is the outcome of interest).

Question 8. Blinding of outcome assessors

Blinding or masking means that the outcome assessors did not know whether the participants received the intervention or were exposed to the factor under study. To answer the question above, the reviewers examined articles for evidence that the person(s) assessing the outcome(s) was masked to the participants' intervention or exposure status. An outcome assessor, for example, may examine medical records to determine the outcomes that occurred in the exposed and comparison groups. Sometimes the person applying the intervention or measuring the exposure is the same person conducting the outcome assessment. In this case, the outcome assessor would not likely be blinded to the intervention or exposure status. A reviewer would note such a finding in the comments section of the assessment tool.

In assessing this criterion, the reviewers determined whether it was likely that the person(s) conducting the outcome assessment knew the exposure status of the study participants. If not, then blinding was adequate. An example of adequate blinding of the outcome assessors is to create a separate committee whose members were not involved in the care of the patient and had no information about the study participants' exposure status. Using a study protocol, committee members would review copies of participants' medical records, which would be stripped of any potential exposure information or personally identifiable information, for prespecified outcomes.

Question 9. Followup rate

Higher overall followup rates are always desirable to lower followup rates, although higher rates are expected in shorter studies, and lower overall followup rates are often seen in longer studies. Usually an acceptable overall followup rate is considered 80 percent or more of participants whose interventions or exposures were measured at baseline. However, this is a general guideline.

In accounting for those lost to followup, in the analysis, investigators may have imputed values of the outcome for those lost to followup or used other methods. For example, they may carry forward the baseline value or the last observed value of the outcome measure and use these as imputed values for the final outcome measure for research participants lost to followup.

Question 10. Statistical analysis

Were formal statistical tests used to assess the significance of the changes in the outcome measures between the before and after time periods? The reported study results should present values for statistical tests, such as p values, to document the statistical significance (or lack thereof) for the changes in the outcome measures found in the study.

Question 11. Multiple outcome measures

Were the outcome measures for each person measured more than once during the course of the before and after study periods? Multiple measurements with the same result increase confidence that the outcomes were accurately measured.

Question 12. Group-level interventions and individual-level outcome efforts

Group-level interventions are usually not relevant for clinical interventions such as bariatric surgery, in which the interventions are applied at the individual patient level. In those cases, the questions were coded as "NA" in the assessment tool.

General Guidance for Determining the Overall Quality Rating of Before-After Studies

The questions in the quality assessment tool were designed to help reviewers focus on the key concepts for evaluating the internal validity of a study. They are not intended to create a list from which to add up items to judge a study's quality.

Internal validity is the extent to which the outcome results reported in the study can truly be attributed to the intervention or exposure being evaluated, and not to biases, measurement errors, or other confounding factors that may result from flaws in the design or conduct of the study. In other words, what is the ability of the study to draw associative conclusions about the effects of the interventions or exposures on outcomes?

Critical appraisal of a study involves considering the risk of potential for selection bias, information bias, measurement bias, or confounding (the mixture of exposures that one cannot tease out from each other). Examples of confounding include co-interventions, differences at baseline in patient characteristics, and other issues throughout the questions above. High risk of bias translates to a rating of poor quality; low risk of bias translates to a rating of good quality. Again, the greater the risk of bias, the lower the quality rating of the study.

In addition, the more attention in the study design to issues that can help determine if there is a causal relationship between the exposure and outcome, the higher quality the study. These issues include exposures occurring prior to outcomes, evaluation of a dose-response gradient, accuracy of measurement of both exposure and outcome, and sufficient timeframe to see an effect.

Generally, when reviewers evaluate a study, they will not see a "fatal flaw," but instead will find some risk of bias. By focusing on the concepts underlying the questions in the quality assessment tool, reviewers should ask themselves about the potential for bias in the study they are critically appraising. For any box checked "no" reviewers should ask, "What is the potential risk of bias resulting from this flaw in study design or execution?" That is, does this factor lead to doubt about the results reported in the study or doubt about the ability of the study to accurately assess an association between the intervention or exposure and the outcome?

The best approach is to think about the questions in the assessment tool and how each one reveals something about the potential for bias in a study. Specific rules are not useful, as each study has specific nuances. In addition, being familiar with the key concepts will help reviewers be more comfortable with critical appraisal. Examples of studies rated good, fair, and poor are useful, but each study must be assessed on its own.

Quality Assessment Tool for Case Series Studies

- Study Quality Assessment Tools

Background: Development and Use

- Study Quality Assessment Tools

Last updated: July, 2021
