# Supplementary material for: Impact of joint commission international accreditation on occupational health and patient safety: A systematic review
Source: PLoS One. 2025 Jun 17;20(6):e0325894. doi: 10.1371/journal.pone.0325894 (PMC12173381; doi:10.1371/journal.pone.0325894)
Supplement: S3 File — (PDF) [file pone.0325894.s003.pdf]

Appendix 2 – Risk of Bias

Risk of bias for interrupted time series studies

Seven standard criteria are used for all interrupted time series studies. Further information can be obtained from Chapter 25: Risk of bias in non-randomized studies of the Cochrane handbook.

Note: If the interrupted time series study has ignored secular (trend) changes and performed a simple *t-test* of the pre versus post intervention periods without further justification, the study should not be included in the review unless reanalysis is possible.

Intervention independent of other changes

Score “Low risk” if there are compelling arguments that the intervention occurred independently of other changes over time and the outcome was not influenced by other confounding variables/historic events during study period. *If Events/variables identified, note what they are.* Score “High risk” if reported that intervention was not independent of other changes in time.

Shape of the intervention effect pre-specified

Score “Low risk” if point of analysis is the point of intervention OR a rational explanation for the shape of intervention effect was given by the author(s). Where appropriate, this should include an explanation if the point of analysis is NOT the point of intervention. Score “High risk” if it is clear that the condition above is not met.

Intervention unlikely to affect data collection

Score “Low risk” if reported that intervention itself was unlikely to affect data collection (for example, sources and methods of data collection were the same before and after the intervention); Score “High risk” if the intervention itself was likely to affect data collection (for example, any change in source or method of data collection reported).

Knowledge of the allocated interventions adequately prevented during the study3,4

4 If some primary outcomes were assessed blindly or affected by missing data and others were not, each primary outcome can be scored separately.

Score “Low risk” if the authors state explicitly that the primary outcome variables were assessed blindly, or the outcomes are objective, e.g. length of hospital stay. Primary outcomes are those variables that correspond to the primary hypothesis or question as defined by the authors. Score “High risk” if the outcomes were not assessed blindly. Score “Unclear risk” if not specified in the paper.

Suggested citation: Cochrane Effective Practice and Organisation of Care (EPOC). [Title]. EPOC Resources for review authors, 2017. epoc.cochrane.org/resources/epoc-resources-review-authors (accessed DD Month YYYY)
