# Supplementary material for: Impact of joint commission international accreditation on occupational health and patient safety: A systematic review
Source: PLoS One. 2025 Jun 17;20(6):e0325894. doi: 10.1371/journal.pone.0325894 (PMC12173381; doi:10.1371/journal.pone.0325894)
Supplement: S4 File — (PDF) [file pone.0325894.s004.pdf]

ID	Study	Included?	Reason for exclusion
1	Anononymous. Quality assurance and accreditation. Q.A. brief, 1997. 6(1): p. 17-19.	No	No follow-up time
2	Anonymous. Joint Commission International Accreditation Standards for Hospitals. Central Lines, 2002. 18(6): p. 10-10	No	No follow-up time
3	Anonymous. Apollo Hospitals installing Rs 30-cr scanning device. Businessline, 2004: p. 1.	No	Excluded after screening title
4	Anononymous. Announcing the new Joint Commission International Center for Patient Safety. Joint Commission perspectives. Joint Commission on Accreditation of Healthcare Organizations, 2005. 25(4): p. 1-2.	No	Excluded after screening abstract
5	Anonymous. Experiences of a hospital in Thailand in treating tsunami patients. World hospitals and health services : the official journal of the International Hospital Federation, 2005. 41(3): p. 24-26.	No	Excluded after screening title
6	Anonymous. Joint Commission International Center for Patient Safety offers new web site. Joint Commission perspectives. Joint Commission on Accreditation of Healthcare Organizations, 2005. 25(6): p. 1-2	No	Excluded after screening abstract
7	Anonymous. World Health Organization partners with Joint Commission and Joint Commission International to eliminate medical errors worldwide. Joint Commission perspectives. Joint Commission on Accreditation of Healthcare Organizations, 2005. 25(10).	No	Excluded after screening abstract
8	Anonymous. Announcement. Joint Commission International Center for Patient Safety offers new Web site. Clinical Nurse Specialist: The Journal for Advanced Nursing Practice, 2005. 19(4): p. 220-220.	No	Excluded after screening abstract
9	Anonymous. Joint Commission International Center for Patient Safety announces new Web site. Medical Malpractice Law & Strategy, 2005. 22(8): p. 11-11.	No	Excluded after screening abstract
10	Anonymous. St. Luke's to build P6.5-B hospital. BusinessWorld, 2005: p. 1.	No	Excluded after screening title
11	Anonymous. Wockhardt Hospitals plans Rs 500-cr expansion: Gets JCI accreditation from US. Businessline, 2005: p. 1.	No	Excluded after screening abstract
12	Anonymous. Apollo to set up hospitals in 4 countries. Businessline, 2006: p. 1.	No	Excluded after screening title
13	Anonymous. Joint Commission International Center for Patient Safety launches new patient safety practices online resource. Nursing News, 2006. 30(3): p. 20-20.	No	Excluded after screening abstract
14	Anonymous. WEEKENDER: health: [1]. BusinessWorld, 2006: p. 1.	No	Excluded after screening title
15	Anonymous. Joint Commission International celebrates a milestone. Bulletin of the American College of Surgeons, 2010. 95(2): p. 36-37	No	Excluded after screening abstract

16	Anonymous. Joint Commission International accreditation standards for hospitals, 4th ed. Reference and Research Book News, 2011. 26(4).	No	Excluded after screening abstract
17	Anonymous. Joint Commission international accreditation; getting started, 2d ed. Reference and Research Book News, 2011. 26(5).	No	Excluded after screening abstract
18	Anonymous. Joint Commission international accreditation; getting started, 2d ed. Reference and Research Book News, 2011. 26(5).	No	Excluded after screening abstract
19	Anonymous. The Inquiry of International Standards for Medical Tourism: A Case Study into Hospitals of Tehran University of Medical Sciences. 2014, Tarbiat Modares University Press. p. 45-50.	No	Excluded after screening abstract
20	Anonymous. Ramesh Hospitals Becomes 1st JCI Accredited Hospital in Andhra. Indian Practitioner, 2020. 73(2): p. 55-55.	No	Excluded after screening abstract
21	Al Shawan, DS. The impact of international accreditation on the quality of health services at King Fahd university hospital, Saudi Arabia: A mixed methods approach	No	No original article
22	Ala, A. et al. Designing a model for medical documentation as per joint commission international in emergency department of Tabriz Imam Reza hospital. Research Journal of Applied Sciences, 2014. 9(8): p. 543-548.	No	Excluded after screening abstract
23	Albanese, J., et al. Fostering disaster resilient communities across the globe through the incorporation of safe and resilient hospitals for community-integrated disaster responses. Prehospital and Disaster Medicine, 2008. 23(5): p. 385-390.	No	Excluded after screening title
24	Algahtani, H., et al. Perception of hospital accreditation among health professionals in Saudi Arabia. Annals of Saudi medicine, 2017. 37(4): p. 326-332.	No	Cross-sectional Study
25	Alimohammadzadeh, K., M. Bahadori, and F. Hassani, Application of Analytical Hierarchy Process Approach for Service Quality Evaluation in Radiology Departments: A Cross-Sectional Study. Iranian journal of radiology : a quarterly journal published by the Iranian Radiological Society, 2016. 13(1): p. e29424-e29424.	No	Excluded after screening title
26	Al-Khayat, H., et al., Risk Factors for Wound Complication in Pilonidal Sinus Procedures. Journal of the American College of Surgeons, 2007. 205(3): p. 439-444.	No	Excluded after screening title
27	Almutairi, A.M., K. Salonitis, and A. Al-Ashaab. A framework for implementing lean principles in the supply chain management at health-care organizations: Saudi's perspective. International Journal of Lean Six Sigma, 2019. 11(3): p. 463-492.	No	Excluded after screening abstract

28	Almutairi, A.M., K. Salonitis, and A. Al-Ashaab. Assessing the leanness of a supply chain using multi-grade fuzzy logic: a health-care case study. International Journal of Lean Six Sigma, 2019. 10(1): p. 81-105.	No	Excluded after screening title
29	Alolayyan, M.N., K.A.M. Ali, and F. Idris. The influence of operational flexibility on hospital performance results: A structural equation modelling (SEM) approaches. International Journal of Services and Operations Management, 2012. 13(4): p. 478-497.	No	Excluded after screening abstract
30	Alsyouf, W.S., et al. Nurses' and Patients' Perceptions of the Quality of Psychiatric Nursing Care in Jordan. Research & Theory for Nursing Practice, 2018. 32(2): p. 226-238.	No	No JCI Accreditation
31	Alves, M.B., et al. Patterns of Care and Temporal Trends in Ischemic Stroke Management: A Brazilian Perspective. Journal of Stroke and Cerebrovascular Diseases, 2017. 26(10): p. 2256-2263.	No	Excluded after screening title
32	Amaral, C., et al. The postoperative venous thromboembolism (TREVO) study – risk and case mortality by surgical specialty. Revista Portuguesa de Cardiologia, 2017. 36(9): p. 609-616.	No	Excluded after screening title
33	Amerioun, A., et al. Assessment of International Joint Commission (IJC) accreditation standard in a military hospital laboratory. Journal of Military Medicine, 2011. 13(2): p. 75-80.	No	No JCI Accreditation
34	Amin, AN. and Owen MM. Productive interdisciplinary team relationships: The hospitalist and the case manager. Lippincott's Case Management, 2006. 11(3): p. 160-164.	No	Excluded after screening title
35	Anonymous. Chapter 1: Industry Profile - 1.1 Asia-Pacific overview. 2011, Emerging Markets Direct Media Holdings, LLC: Beverly. p. 1-3.	No	Excluded after screening title
36	Asché, P. Accreditation of hospitals in the USA according to the standards of the Joint Commission on Accreditation of Healthcare Organisations - Considerations about a transfer of this process on Germany. Zeitschrift fur Arztliche Fortbildung und Qualitatssicherung, 2000. 94(8): p. 665-668.	No	Excluded after screening abstract
37	Avia, I. and R.T.S. Hariyati. Impact of hospital accreditation on quality of care: A literature review. Enfermeria Clinica, 2019. 29: p. 315-320.	No	Excluded after screening abstract
38	Bah, S. and Alanzi T. Comparison of three internship training sites for an undergraduate health information management program in Saudi Arabia. Healthcare Informatics Research, 2017. 23(3): p. 233-237.	No	Excluded after screening title

39	Bahadori, M., et al. Assessing the service quality of Iran military hospitals: Joint Commission International standards and Analytic Hierarchy Process (AHP) technique. Journal of Education and Health Promotion, 2014. 3: p. 98.	No	Excluded after screening abstract
40	Bahcecik N. and Ozturk H. The Occupational Safety and Health in Hospitals from the Point of Nurses. Collegium Antropologicum, 2009. 33(4): p. 1205-1214.	No	No JCI Accreditation
41	Ballerio, S. Automatic analysis of electronic discharge letters as a means to evaluate the continuity of information and of patient care. Proceedings of the 13th International Conference on Electronic Publishing (ELPUB 2009), 2009: p. 109–120. DOI: 10.3233/978-1-60750-028-5-109.	No	Excluded after screening abstract
42	Banjar A., et al. Surveillance of preventive measures for ventilator associated pneumonia (VAP) and its rate in Makkah Region hospitals, Saudi Arabia. Turkish Journal of Medical Sciences, 2017. 47(1): p. 211-216.	No	Excluded after screening title
43	Banzon-Natad J. Cebu positioning to compete in medical tourism. BusinessWorld, 2008: p. 1.	No	Excluded after screening title
44	Bar-Ratson E., et al. The accreditation program in hospitals: Clalit Health Services experience]. Harefuah, 2011. 150(4): p. 340-345, 420, 419.	No	Excluded after screening abstract
45	Bener, A. and A. Al Mazroei. Health services management in Qatar. Croatian medical journal, 2010. 51(1): p. 85-88.	No	Excluded after screening abstract
46	Bianchi N. Nursing documentation at the Bambino gesù children's hospital. Paediatrics and Child Health, 2008. 18(SUPPL. 1): p. S80-S83.	No	No JCI Accreditation
47	Bianchi N., Carta G. and T. Renzetti. Clinical risk management at the Bambino Gesù Children's Hospital. Paediatrics and Child Health, 2009. 19(SUPPL. 2): p. S176-S181.	No	No JCI Accreditation
48	Bobadilla CC. SPECIAL FEATURE: Modern Medical Centers: [3]. BusinessWorld, 2007: p. 1.	No	Excluded after screening title
49	Bogaert PV et al., Staff empowerment and engagement in a magnet® recognized and joint commission international accredited academic centre in Belgium: a cross-sectional survey. BMC health services research, 2018. 18(1): p. 756-756.	No	Cross-sectional Study
50	Bogh SB., et al. Accreditation and improvement in process quality of care: A nationwide study. International Journal for Quality in Health Care, 2015. 27(5): p. 336-343.	No	No JCI Accreditation
51	Bohigas L. Accreditation across borders: the introduction of Joint Commission accreditation in Spain. The Joint Commission journal on quality improvement, 1998. 24(5): p. 226-231.	No	Excluded after screening abstract

52	Bosio M. and Meroni P. Quality and efficiency of the Health Service in the Italian Region of Lombardy. Giornale italiano di nefrologia : organo ufficiale della Società italiana di nefrologia, 2002. 19 Spec No 21: p. S28-32.	No	Excluded after screening abstract
53	Bressan R., et al. Spread of vancomycin-resistant enterococcus faecium isolates despite validated infection control measures in an Italian Hospital: Antibiotic resistance and genotypic characterization of the endemic strain. Microbial Drug Resistance, 2018. 24(8): p. 1148-1155.	No	Excluded after screening title
54	Buffoli M., et al. Healthcare sustainability evaluation systems, in Green Energy and Technology. 2015. p. 23-29.	No	Excluded after screening abstract
55	Burns GB. and Hogue V. WellStar paulding hospital intensive care unit case study: Achieving a research-based, patient-centered design using a collaborative process. Critical Care Nursing Quarterly, 2014. 37(1): p. 93-102.	No	Excluded after screening abstract
56	Buzzi F. Information and consent for the purposes of an appropriate medico-legal management of nosocomial infections, especially in light of the indications by the joint commission international accreditation and addresses of the supreme court. Giornale Italiano di Medicina del Lavoro ed Ergonomia, 2010. 32(3): p. 312-317.	No	Excluded after screening abstract
57	Cagoco JL. Integration seen key to competing. BusinessWorld, 2006: p. 1.	No	Excluded after screening title
58	Carta, G., N. Bianchi, and M. Pomponi. Quality of nursing care at Bambino Gesù Children's Hospital. Paediatrics and Child Health, 2009. 19(SUPPL. 2): p. S168-S171.	No	No follow-up time
59	Cato DL., et al. The CNO US Healthcare Immersion Program, Part 1: A Transformational Leadership Model. Nursing Administration Quarterly, 2019. 43(1): p. 40-49.	No	Excluded after screening title
60	Cebeci U. Joint commission international consultant selection for hospitals by fuzzy AHP. 2008	No	Excluded after screening abstract
61	Cengi C., Celik Y., and Neset H. Evaluation of patient wristbands and patient identification process in a training hospital in Turkey. International Journal of Health Care Quality Assurance, 2016. 29(8): p. 820-834.	No	Excluded after screening title
62	Chang, CH., Lai YL. and Chen CC. Implement the RFID position based system of automatic tablets packaging machine for patient safety. Journal of Medical Systems, 2012. 36(6): p. 3463-3471.	No	Excluded after screening abstract

63	Chen M. and Zhou Q. Pharmaceutical interventions by collaboration between staff pharmacists and clinical pharmacists and implementation of Joint Commission International Accreditation Standards on medication use may optimize pharmacotherapy in geriatric patients. Clinical interventions in aging, 2016. 11: p. 1575-1577.	No	Excluded after screening abstract
64	Chen QL., et al. Retrospective analysis of non-laboratory-based adverse drug reactions induced by intravenous radiocontrast agents in a Joint Commission International-accredited academic medical center hospital in China. Therapeutics and clinical risk management, 2017. 13: p. 565-573.	No	Excluded after screening title
65	Chung, O. Tourism for the health of it. Taiwan Review, 2008. 58(2): p. 12-17.	No	Excluded after screening title
66	Corrêa AdG., et al. Analysis Treatment Guideline versus Clinical Practice Protocol in Patients Hospitalized due to Heart Failure. Arquivos brasileiros de cardiologia, 2016. 106(3): p. 210-217	No	Excluded after screening abstract
67	Sá-Correia L, Correia ME, Cruz-Correia R. Illegitimate HIS Access by Healthcare Professionals Detection System Applying an Audit Trail-based Model. In: Proceedings of the 13th International Joint Conference on Biomedical Engineering Systems and Technologies (BIOSTEC 2020) - HEALTHINF. 2020:539–546. DOI: 10.5220/0008991505390546	No	Excluded after screening abstract
68	Creixans-Tenas J., Coenders G. and Arimany-Serrat N. Corporate social responsibility and financial profile of Spanish private hospitals. Heliyon, 2019. 5(10).	No	Excluded after screening title
69	Dackiewicz N., et al. Health care team and parents opinion about pediatric patients identification. Archivos Argentinos de Pediatria, 2011. 109(2): p. 105-110.	No	Excluded after screening abstract
70	Davins J., et al. Catalonia's primary healthcare accreditation model: A valid model. Medicina Clinica, 2014. 143(SUPPL. 1): p. 74-80.	No	Excluded after screening abstract
71	Day SW. Evaluating the impact of the Guatemalan Nursing Program on staff, organizational, and clinical outcomes. 2010, University of Tennessee Health Science Center. p. 93 p-93 p	No	Excluded after screening abstract
72	Day SW., et al. Quality assessment of pediatric oncology nursing care in a Central American country: findings, recommendations, and preliminary outcomes. Pediatric nursing, 2008. 34(5): p. 367-373.	No	Excluded after screening abstract

73	Day SW., et al. Use of Joint Commission International standards to evaluate and improve pediatric oncology nursing care in Guatemala. Pediatric Blood & Cancer, 2013. 60(5): p. 810-815.	No	No JCI Accreditation
74	De Grande RS. and De Mendes GHS. Impacts of Joint Commission International Hospital Accreditation in a Brazilian hospital. Espacios, 2015. 36(20): p. 10.	No	Language other than English
75	De Meester K., et al. SBAR improves nurse-physician communication and reduces unexpected death: A pre and post intervention study. Resuscitation, 2013. 84(9): p. 1192-1196.	No	No JCI Accreditation
76	Debata BR., et al. An integrated approach for service quality improvement in medical tourism: An Indian perspective. International Journal of Services and Operations Management, 2012. 13(1): p. 119-145.	No	No JCI Accreditation
77	Deriu PL., et al. OECI accreditation of the European Institute of Oncology of Milan: Strengths and weaknesses. Tumori, 2015. 101: p. S21-S24.	No	Excluded after screening abstract
78	Despotou G., Her J. and Arvanitis TN. Nurses' Perceptions of Joint Commission International Accreditation on Patient Safety in Tertiary Care in South Korea: A Pilot Study. Journal of Nursing Regulation, 2020. 10(4): p. 30-36.	No	Cross-sectional Study
79	Dewan S., et al. The Apollo Accreditation Program: A web-based Joint Commission International standards compliance management tool. World hospitals and health services : the official journal of the International Hospital Federation, 2014. 50(4): p. 31-34.	No	Excluded after screening abstract
80	Devkaran S. and O'Farrell PN. The impact of hospital accreditation on clinical documentation compliance: a life cycle explanation using interrupted time series analysis. BMJ Open, 2014. 4(8).	Yes	
81	Devkaran S. and O'Farrell PN. The impact of hospital accreditation on quality measures: An interrupted time series analysis Quality, performance, safety and outcomes. BMC Health Services Research, 2015. 15(1).	Yes	
82	Devkaran S., et al. Impact of repeated hospital accreditation surveys on quality and reliability, an 8-year interrupted time series analysis. BMJ Open, 2019. 9(2): p. 1V.	Yes	
83	Dhatt GS. and Al Sheiban A. Joint Commission International accreditation: a laboratory perspective. Accreditation and Quality Assurance, 2008. 13(3): p. 161-164.	No	Excluded after screening abstract
84	Donahue KT. and Vanostenberg P. Joint Commission International accreditation: Relationship to four models of evaluation. International Journal for Quality in Health Care, 2000. 12(3): p. 243-246.	No	No follow-up time

85	Donahue KT. and Yen J. Joint Commission International. Joint Commission Journal on Quality Improvement, 1997. 23(1): p. 71-71	No	No follow-up time
86	Dotta A., et al. Accreditation of birth centres: Advantages for newborns. Journal of Maternal-Fetal and Neonatal Medicine, 2013. 26(4): p. 417-418.	No	No follow-up time
87	Esler D. Protecting Crew and Passengers From Health Hazards When Flying Abroad. Business & Commercial Aviation, 2014: p. 26-28,32,34-35.	No	Excluded after screening title
88	Esperanza QP. Study of medical oxygen use in chronic obstructive pulmonary disease according to tracer methodology of joint commission. European Journal of Clinical Pharmacy, 2017. 19(3): p. 190-197.	No	Excluded after screening title
89	Anonymous. Health City Cayman Island. Consulting - Specifying Engineer, 2015	No	Excluded after screening title
90	Fanelli S., Ferretti M. and Zangrandi A. The impact of regional policies on emergency department management and performance: the case of the regional government of Sicily. The International Journal of Health Planning & Management, 2017. 32(1): p. e83-e98.	Yes	
91	Farzianpour F., et al. Assessment of quality of life in cancer patients. American Journal of Agricultural and Biological Science, 2014. 9(2): p. 147-152.	No	Excluded after screening title
92	Fatima N., et al. Discordant interpretation of serial bone mineral density measurements by dual-energy X-ray absorptiometry using vendor's and institutional least significant changes: Serious impact on decision-making. World journal of nuclear medicine, 2018. 17(4): p. 236-240.	No	Excluded after screening title
93	Feng XQ., Zhu LL. and Zhou Q. The checking methods before medication administration: A perspective from a Joint Commission International–accredited academic medical center hospital in China. Journal of Evaluation in Clinical Practice, 2017. 23(3): p. 676-678.	No	No JCI Accreditation
94	Fernández-Castelló AI., et al. An experience in integrated management of clinical risks. Journal of Healthcare Quality Research, 2018. 33(6): p. 311-318.	No	No JCI Accreditation
95	Folkerts V. and Lipson R. United family healthcare (chindex international): A case study, in China's Healthcare System and Reform. 2017. p. 250-268.	No	Excluded after screening title
96	Franco-Clark D., Pimentel-Aguilar AB. and Rodríguez-Vera R. Design of a medical and laboratory equipment management program for the new standards certification achievement in Mexico. 2010.	No	Excluded after screening abstract

97	Furukawa PdO. and Cunha ICKO. Profile and Competencies of Nurse Managers at Accredited Hospitals. Revista Latino-Americana de Enfermagem (RLAE), 2011. 19(1): p. 106-114.	No	Excluded after screening abstract
98	Galatama Purwadi A., Sulistiadi W. and Asyary A. Understanding Implementation of Patient Safety Goals Framework at Inpatient Unit of Ciracas General Hospital, Indonesia. 2019.	No	No JCI Accreditation
99	Galván-García ÁF., et al. Results of certification audit in Mexican hospitals, a review from 2009 to 2012. Salud Publica de Mexico, 2018. 60(2): p. 202-211.	No	Excluded after screening abstract
100	Ganapathy K., et al. Tele-Emergency Services in the Himalayas. Telemedicine and e-Health, 2019. 25(5): p. 380-390.	No	Excluded after screening title
101	Gillentine A. Colorado Springs Medical Briefs: December 15, 2006. The Colorado Springs Business Journal (Pre- June 2, 2012), 2006: p. 1.	No	Excluded after screening title
102	Gillentine A. Blue Cross and Blue Shield of South Carolina creates relationship with Thai hospital. The Colorado Springs Business Journal (Pre- June 2, 2012), 2007: p. 1.	No	Excluded after screening title
103	Gingerich BS. Accreditation and licensing actions. The Joint Commission International Center for Patient Safety. Home Health Care Management & Practice, 2006. 18(5): p. 418-419.	No	No JCI Accreditation
104	Gong S., et al. Effect of Financially Punished Audit and Feedback in a Pediatric Setting in China, within an Antimicrobial Stewardship Program, and as Part of an International Accreditation Process. Frontiers in public health, 2016. 4: p. 99-99.	No	No JCI Accreditation
105	Gong XY., et al. A rapid response team is associated with reduced overall hospital mortality in a Chinese tertiary hospital: a 9-year cohort study. Annals of translational medicine, 2020. 8(6): p. 317-317.	No	Excluded after screening abstract
106	Halasa YA., et al. Value and impact of international hospital accreditation: a case study from Jordan. Eastern Mediterranean Health Journal, 2015. 21(2): p. 90-99.	Yes	
107	Hashemi B., et al. An Audit of Emergency Department Accreditation Based on Joint Commission International Standards (JCI). Emergency (Tehran, Iran), 2014. 2(3): p. 130-133.	No	No JCI Accreditation
108	Hashim NH. Computer Guided Hospital Accreditation Management System. 2018.	No	Excluded after screening abstract
109	Hassan D. Measuring performance in the healthcare field: A multiple stakeholders' perspective. Total Quality Management & Business Excellence, 2005. 16(8,9): p. 945-954.	No	No JCI Accreditation

110	Hawkins R. Managing the pre- and post-analytical phases of the total testing process. Annals of Laboratory Medicine, 2012. 32(1): p. 5-16.	No	Excluded after screening title
111	Hijjeh M., et al. Critical Care Network in the State of Qatar. Qatar medical journal, 2019. 2019(2): p. 2-2.	No	Excluded after screening title
112	Hoe J. Quality service in radiology. Biomedical Imaging and Intervention Journal, 2007. 3(3	No	Excluded after screening abstract
113	Inal TC. Joint Commission International Accreditation for Clinical Laboratories: Monitor, analyze and improve. Clinical Biochemistry, 2009. 42(4-5): p. 303	No	Not original article
114	Inomata T., et al. The impact of Joint Commission International accreditation on time periods in the operating room: A retrospective observational study. PLoS ONE, 2018. 13(9).	Yes	
115	Inworn N. and Chompu-Inwai R. Factors for assessing performance of lean concept application in hospital outpatient department. 2015.	No	Excluded after screening abstract
116	Iravan Masoudi A., et al. Relationship between Health Care Organization Management Standards of the Joint Commission International and Health Tourism in Selected Hospitals in Tehran. International Journal of Travel Medicine & Global Health, 2014. 2(1): p. 19-22.	No	Excluded after screening abstract
117	Jaber H. The Impact of Accreditation on Quality of Care: Perception of Nurses in Saudi Arabia. 2014, Walden University. p. 151 p-151 p.	No	Not original article
118	Jacquemyn Y. Accreditation and resulting clerical duties represent commercial excesses that are ethically and scientifically unacceptable. Facts, views & vision in ObGyn, 2018. 10(2): p. 59-61	No	Excluded after screening abstract
119	Jafary FH., Ahmed H. and Kiani J. Outcomes of primary percutaneous coronary intervention at a Joint Commission International Accredited hospital in a developing country - Can good results, possibly similar to the west, be achieved? Journal of Invasive Cardiology, 2007. 19(10): p. 417-423.	No	Excluded after screening title
120	Jimeno E. et al. Joint Commission International and implementation in CPR learning. Resuscitation, 2018. 130: p. e70-e70.	No	No JCI Accreditation
121	Johnson MC. et al. SafeCare: An innovative approach for improving quality through standards, benchmarking, and improvement in low-and middle-income countries. Joint Commission Journal on Quality and Patient Safety, 2016. 42(8): p. 350-360.	No	Excluded after screening abstract
122	Juanhua C. and Jinjuan WU. Implementation of appointment scheduling based on Joint Commission International standards for patients undergoing elective cesarean section. Nursing of Integrated Traditional Chinese & Western Medicine, 2019. 5(12): p. 119-121	No	Language other than English

123	Kagan I., Farkash-Fink N. and Fish M. Effect of Joint Commission International Accreditation on the Nursing Work Environment in a Tertiary Medical Center. Journal of Nursing Care Quality, 2016. 31(4): p. E1-E8.	Yes	
124	Kagan I., et al. Computerization and its contribution to care quality improvement: The nurses' perspective. International Journal of Medical Informatics, 2014. 83(12): p. 881-888.	Yes	
125	Kalhor R. et al. Evaluation of the quality of services delivered in qazvin's hospitals to attract medical tourists: Joint commission international approach. Journal of Biology and Today's World, 2016. 5(12): p. 224-229.	No	Excluded after screening abstract
126	Karim M., et al. Postgraduate Training in a Low- and Middle-income Country: Sharing Experience from a Joint Commission International Accreditation-accredited University Hospital. Clinical Oncology, 2017. 29(9): p. 636-637	No	Excluded after screening abstract
127	Khan S., et al. SAU: A potential destination for medical tourism. Journal of Taibah University Medical Sciences, 2014. 9(4): p. 257-262.	No	Excluded after screening title
128	Kilinc C. Laboratory quality management systems: Missions, goals and activities in quality assurance. Clinical Biochemistry, 2009. 42(4-5): p. 301-302	No	Excluded after screening abstract
129	Kim Y. e. a. SMART Careplan System for Continuum of Care. Healthcare informatics research, 2015. 21(1): p. 56-60	No	Excluded after screening abstract
130	Kulkarni M. Understanding reasons for rise in conflict situation in an Indian hospital and suggesting measures to minimize them. Indian Journal of Public Health Research and Development, 2019. 10(7): p. 1566-1570.	No	Excluded after screening title
131	Kurtulmuş S., et al. The rise of medical tourism in Turkey and the unique case of patients from Germany, in Turkish German Affairs from an Interdisciplinary Perspective. 2015. p. 323-352.	No	Excluded after screening title
132	Kuster G. Performance of four ischemic stroke prognostic scores in a Brazilian population. Arquivos de Neuro-Psiquiatria, 2016. 74(2): p. 133-137.	No	Excluded after screening title
133	Kweon T. Sedation under JCI standard. Korean Journal of Anesthesiology, 2011. 61(3): p. 190-194	No	No JCI Accreditation
134	Kweon T. Update of sedation in view of Joint Commission International standards. Journal of the Korean Medical Association, 2011. 54(12): p. 1284-1288.	No	Excluded after screening abstract
135	Köse G. The importance of "patient-nursephysician-pharmacist" collaboration on drug administrations. Marmara Pharmaceutical Journal, 2012. 16(2): p. 115-119.	No	Excluded after screening abstract

136	Lalisang T. Clinical Practice Guidelines in Complicated Intra-Abdominal Infection 2018: An Indonesian Perspective. Surgical Infections, 2019. 20(1): p. 83-90.	No	No JCI Accreditation
137	Lang S. Patient's experiences of spiritual care: a phenomenological approach. Singapore Nursing Journal, 2006. 33(3): p. 42-47	No	Excluded after screening abstract
138	Lauwers M. Surgical safety checklist and the joint commission international. Tijdschrift voor Geneeskunde, 2018. 74(6): p. 410-417.	No	Excluded after screening title
139	Lazaryan M, Abu-Kishk I, Rosenfeld-Yehoshua N, Berkovitch S, Toledano M, Reshef I, Kanari T, Ziv-Baran T, Berkovitch M. Pharmacist Remote Review of Medication Prescriptions for Appropriateness in Pediatric Intensive Care Unit. Frontiers in Pharmacology, 2016 Aug 9;7:243. DOI: 10.3389/fphar.2016.00243. PMID: 27555821; PMCID: PMC4977293	No	No JCI Accreditation
140	Lee H., et al. The significance of Joint Commission International accreditation. Journal of the Korean Medical Association, 2012. 55(1): p. 17-22.	No	Excluded after screening abstract
141	Liddick B. Going the Distance for Health Savings. HRMagazine, 2007. 52(3): p. 50-55.	No	Language other than English
142	Lili Z. Ventilator bundle guided by context of JCI settings can effectively reduce the morbidity of ventilatorassociated pneumonia. Zhonghua Wei Zhong Bing Ji Jiu Yi Xue, 2017. 29(7): p. 624-628.	No	Excluded after screening abstract
143	Linden A. Dealing With Knowledge Management to Build a Safer Health Care. 2015, Academic Conferences International Limited: Kidmore End. p. 1008-1010.	No	No JCI Accreditation
144	Lopes C. The business model and innovation analyses: The sustainable transition obstacles and drivers for the hospital supply chains. Resources, 2019. 8(1).	No	Excluded after screening title
145	Lovern E. Accreditation gains attention. Modern Healthcare, 2000. 30(47): p. 46.	No	No follow-up time
146	Lundgren B., et al. Infection control standards and accreditation. Ugeskrift for Laeger, 2007. 169(48): p. 4142-4144.	No	No follow-up time
147	Lupieri G. C. C., A. Palese C. Cardio-thoracic surgical patients' experience on bedside nursing handovers: Findings from a qualitative study. Intensive & Critical Care Nursing, 2016. 35: p. 28-37.	No	Excluded after screening abstract
148	Macedo T. Clinical-care protocol for preventing mediastinitis after coronary artery bypass graft surgery: A quality improvement initiative from a private hospital. Journal of Cardiac Surgery, 2019. 34(5): p. 274-278.	No	No follow-up time

149	Massa L. et. al. Indications for cardiology consultation and management of cardiac patients who will undergo surgical or endoscopic procedures: Applicative guidelines by the University Hospital of Trieste, Italy. Giornale Italiano di Cardiologia, 2010. 11(7-8): p. 590-598.	No	Excluded after screening abstract
150	Mehta A. S. G., et al. , Global trends in center accreditation by the Joint Commission International: growing patient implications for international medical and surgical care. Journal of Travel Medicine, 2017. 24(5).	No	No follow-up time
151	Mekory T. The proportion of errors in medical prescriptions and their executions among hospitalized children before and during accreditation. International Journal for Quality in Health Care, 2017. 29(3): p. 366-370.	Yes	
152	Moe J. G. P., et al. Transformational leadership, transnational culture and political competence in globalizing health care services: A case study of Jordan's King Hussein Cancer Center. Globalization and Health, 2007. 3.	No	Excluded after screening title
153	Mohmmad Pour A. The Ministry of Health's Care of Patient Standards vs. Joint Commission International Accreditation Standards for Care of Patient. Journal of Medical Council of Islamic Republic of Iran, 2011. 29(2): p. 191-191.	No	Excluded after screening abstract
154	Mohsenian Sisakht A. e. a. Adherence to informed consent standards in Shiraz hospitals: matrons' perspective. International journal of health policy and management, 2014. 4(1): p. 13-18.	No	Excluded after screening title
155	Moore J. J. Setting standards. Modern Healthcare, 1997. 27(44): p. I32-I33.	No	Excluded after screening abstract
156	Moreno M., et al. Hospital Excellence Operation Model: an Approach to Lean Healthcare in Mexican Hospitals. IIE Annual Conference. Proceedings, 2012: p. 1-10.	No	No follow-up time
157	Nam Young Y., et al. Relationships of Nurses' Perception, Nursing Performance, Job Stress, and Burnout in Relation to the Joint Commission International Hospital Accreditation. Journal of Korean Academy of Nursing Administration, 2014. 20(1): p. 1-9.	No	No follow-up time
158	Ng W, Chow MPY, Wu WQ, Png HH, Jacob E. Sustaining robust practices in point-of-care testing through connectivity. Point of Care, 2009 Sep;8(3):117–120. DOI: 10.1097/POC.0b013e3181b316a8.	No	No follow-up time
159	Nimeri A. J. B., et al. Reducing Healthcare Costs Using ACS NSQIP-Driven Quality Improvement Projects: A Success Story from Sheikh Khalifa Medical City (SKMC). World Journal of Surgery, 2019. 43(2): p. 331-338.	No	No follow-up time

160	Nomura A. M. S., et al. Quality of nursing documentation before and after the Hospital Accreditation in a university hospital. Revista latino-americana de enfermagem, 2016. 24: p. e2813.	No	Language other than English
161	Noparatayaporn P. Economic evaluation of ready-to-use injectable medications by pharmacy department compared with the traditional system of individual preparation by nurse. Siriraj Medical Journal, 2019. 71(1): p. 25-30.	No	Excluded after screening abstract
162	Oguego N. Audit of ophthalmology discharge summaries in a Nigerian Teaching Hospital. Nigerian Journal of Clinical Practice, 2018. 21(7): p. 901-906.	No	Excluded after screening title
163	Okumura L. D. S., et al. Relation between safe use of medicines and Clinical Pharmacy Services at Pediatric Intensive Care Units. Revista paulista de pediatria : orgao oficial da Sociedade de Pediatria de Sao Paulo, 2016. 34(4): p. 397-402	No	Excluded after screening abstract
164	Okumura Y. et. al. Shortened cataract surgery by standardisation of the perioperative protocol according to the Joint Commission International accreditation: a retrospective observational study. BMJ open, 2019. 9(6): p. e028656-e028656.	Yes	
165	O'Leary D. Patient safety: the search for global solutions. World hospitals and health services : the official journal of the International Hospital Federation, 2008. 44(1): p. 19-21.	No	No follow-up time
166	Olvera-Arreola S. Factors relating to falls in hospitalized patients. Revista de Investigacion Clinica, 2013. 65(1): p. 88-93.	No	Excluded after screening abstract
167	Pafford B. The third wave - Medical tourism in the 21st century. Southern Medical Journal, 2009. 102(8): p. 810-813.	No	Excluded after screening title
168	Paradise A., et al. Global Medical Networks Make Medical Travel a Viable Option. Benefits & Compensation Digest, 2007. 44(4): p. 40-43	No	Excluded after screening title
169	Park C. The current state of sedation outside the operating room. Journal of the Korean Medical Association, 2013. 56(4): p. 264-270	No	Excluded after screening abstract
170	Pasternak D. Bedside (point-of-care) testing in hospitals: The joint commission international perspective. Point of Care, 2008. 7(4): p. 233-238.	No	No follow-up time
171	Poe S., et al. Joint commission international center for patient safety web site: A critical appraisal. Journal of Nursing Care Quality, 2006. 21(2): p. 114-118.	No	No follow-up time
172	Pravisani R. e. a. Does the status of surgical resident compared to that of consultant have an impact on patient's satisfaction over the informed consent process? Annali Italiani di Chirurgia, 2018. 89(2): p. 182-189.	No	Excluded after screening abstract

173	Qarari, H. Analyzing patient satisfaction towards resident at internal medicine department in Hasan Sadikin Hospital Bandung using revised importance–performance analysis. International Journal of Business and Society, 2018. 19(1): p. 59–72.	No	Excluded after screening abstract
174	Qi J. Y. H., et al. Experience of improving the quality of pharmaceutical care based on the Joint Commission International Accreditation. Pharmaceutical Care and Research, 2014. 14(2): p. 158-160. .	No	Excluded after screening abstract
175	Quintaliani G. Chronic kidney disease certification process manual by the Italian Society of Nephrology (SIN): Part II: Programme management and clinical information management. Journal of Nephrology, 2009. 22(5): p. 565-570.	No	Language other than English
176	Reuther F. Avoidance of wrong site surgery : EEEExperiences by the introduction of measures for quality control and patient safety in a surgical casualty hospital. Unfallchirurg, 2009. 112(7): p. 675-678.	No	Excluded after screening title
177	Revathi J., et al. Analysis of medical tourism and its economic impact. Indian Journal of Public Health Research and Development, 2019. 10(1): p. 21-25.	No	Language other than English
178	Rhea S. Global project. Joint commission international starts quality, safety demo in three locations. Modern healthcare, 2008. 38(42): p. 17	No	No follow-up time
179	Rienzi L. Comprehensive protocol of traceability during IVF: The result of a multicentre failure mode and effect analysis. Human Reproduction, 2017. 32(8): p. 1612-1620.	No	Excluded after screening title
180	Rizzi F. Quality for home palliative care: An Italian metropolitan multicentre JCI-certified model. BMJ Quality and Safety, 2011. 20(7): p. 592-598.	No	No follow-up time
181	Rossi, L. A web-based application with digital signature for drugs dispensing management. Published in Studies in Health Technology and Informatics, 2006. 121: p. 217–224. DOI: 10.3233/978-1-58603-637-4-217	No	Excluded after screening title
182	Sainul Abideen P. Practical implications of spontaneous adverse drug reaction reporting system in hospitals - An overview. Asian Journal of Pharmaceutical and Clinical Research, 2013. 6(4): p. 10-15.	No	Excluded after screening title
183	Santos R. L. P., et al. Naming babies "Baby-of" at birth: A project to encourage earlier naming of infants in Abu Dhabi. BMJ quality improvement reports, 2015. 4(1): p. u207266.w2927.	No	Excluded after screening title

184	Shafaghat T. The Capabilities of Iranian Hospitals in Attracting Medical Tourists; Based on Joint Commission International: A Case Study of Shiraz Hospitals. International Journal of Travel Medicine & Global Health, 2014. 2(1): p. 5-9.	No	Excluded after screening abstract
185	Shaffer A., et al. First Chinese public hospital wins Joint Commission International accreditation: the Health Information Department played a key role in the process. Journal (Institute of Health Record & Information Management : 2008), 2008. 49(2): p. 51-53.	No	Excluded after screening abstract
186	Sharifi M. e. a. Developing a Model for Accreditation of Iranian Teaching Hospitals: A Qualitative Study. Ethiopian journal of health sciences, 2019. 29(6): p. 657-668.	No	Excluded after screening abstract
187	.Shum A. Securement of the indwelling urinary catheter for adult patients: a best practice implementation. International Journal of Evidence-Based Healthcare, 2017. 15(1): p. 3-12.	No	Excluded after screening title
188	Sim W. e. a. Scope of Hand Surgery Using Surgeon Administered Local/Regional Anaesthesia. Annals of Plastic Surgery, 2019. 83(3): p. 278-284.	No	Excluded after screening title
189	Song P. W. L., et al. An outpatient antibacterial stewardship intervention during the journey to JCI accreditation. BMC pharmacology & toxicology, 2014. 15: p. 8-8.	Yes	
190	Steenbruggen R. Development of quality indicators for departments of hospital-based physiotherapy: a modified Delphi study. BMJ open quality, 2020. 9(2): p. e000812.	No	Excluded after screening abstract
191	Suharjono and Ardianto. Making high-risk medication safer to use. Journal of Pharmacy Practice & Research, 2018. 48(6): p. 501-503.	No	No follow-up time
192	Tan H. T. S., et al. Quality improvement: experience of a sexually transmitted infection clinic in Singapore. International Journal of STD & AIDS, 2008. 19(12): p. 800-804.	No	Excluded after screening abstract
193	Tavakoli N., et al. External Evaluation of Four Hospitals According to Patient-centred Care Standards. Acta Informatica Medica, 2013. 21(3): p. 176.	No	Excluded after screening abstract
194	Terzioglu F. S. T., et al. Factors affecting performance and productivity of nurses: professional attitude, organisational justice, organisational culture and mobbing. Journal of Nursing Management, 2016. 24(6): p. 735-744.	No	Excluded after screening abstract
195	Thakur S. Corrosion of the Code of Medical Ethics. Medico-Legal Update, 2016. 16(2): p. 69-74.	No	Excluded after screening abstract

196	Truzyan N. e. a. Quality of Inpatient Tuberculosis Health Care in High-Burden Resource-Limited Settings: Protocol for a Comprehensive Mixed Methods Assessment Study. JMIR research protocols, 2020. 9(1): p. e13903-e13903	No	Excluded after screening abstract
197	Truzyan N.Bridging the gap between international standards of quality of care and practices in the inpatient unit of the National TB Control Center in Armenia. Value in Health: The Journal of the International Society for Pharmacoeconomics and Outcomes Research, 2017. 20(9).	No	Excluded after screening abstract
198	Tsao S., et al. Pain management experience at a central Taiwan medical center. Acta Anaesthesiologica Taiwanica, 2015. 53(2): p. 66-70.	No	Excluded after screening abstract
199	Ueshima H. N. K., et al. Hospital doctors should receive lecture for sedation according to joint commission international. Japanese Journal of Anesthesiology, 2015. 64(8): p. 883-887.	No	Excluded after screening abstract
200	Um M. International hospital accreditation and clinical nutrition service in acute care hospitals in South Korea: Results of a nationwide cross-sectional survey. Asia Pacific Journal of Clinical Nutrition, 2018. 27(1): p. 158-166.	No	Cross-sectional study
201	van der Leij S, Schneider MME, Geerlings SE, Kaasjager KAH. Tien tips voor het optimaliseren van de overdracht. Nederlands Tijdschrift voor Geneeskunde, 2015;159:A9085	No	Excluded after screening title
202	Wang B. Practice of continuous quality improvement in rational application of antibacterials by the joint commission international evaluation. Pharmaceutical Care and Research, 2014. 14(3): p. 195-198.	No	No follow-up time
203	Wang H. Quality improvements in decreasing medication administration errors made by nursing staff in an academic medical center hospital: A trend analysis during the journey to Joint Commission International accreditation and in the post-accreditation era. Therapeutics and Clinical Risk Management, 2015. 11: p. 393-406.	Yes	
204	Vanostenberg P. Quality measurement across borders: needs and options. World hospitals and health services : the official journal of the International Hospital Federation, 2006. 42(1): p. 19-22.	No	No follow-up time
205	Warfield JE. Get to Know JCAHO and Its Standards. Medical Design News, 2006; p. 78–79.	No	Excluded after screening title
206	Wilkins S. Functional Stroke Mimics: Incidence and Characteristics at a Primary Stroke Center in the Middle East. Psychosomatic Medicine, 2018. 80(5): p. 416.	No	Excluded after screening title

207	Virginio L., et al. Finding Relations Between Requirements for Healthcare Information Systems Use in Hospitals: A Study on EMRAM and JCI. 2019.	No	Excluded after screening abstract
208	Woodhead A. Scoping medical tourism and international hospital accreditation growth. International Journal of Health Care Quality Assurance, 2013. 26(8): p. 688-702.	No	No follow-up time
209	Wu S. Using Plan-Do-Check-Act Circulation to Improve the Management of Panic Value in the Hospital. Chinese Medical Journal, 2015. 128(18).	No	Excluded after screening abstract
210	Xu F. Et al. Safe medication management and use of narcotics in a Joint Commission International-accredited academic medical center hospital in the People's Republic of China. Therapeutics and Clinical Risk Management, 2016. 12: p. 535-544.	No	No follow-up time
211	Yang M. The experience of improving rapid response system performance in a Chinese joint commission international hospital. Biomedical Research (India), 2017. 28(19): p. 8215-8220.	No	No follow-up time
212	Yang M. Improving rapid response system performance in a Chinese Joint Commission International Hospital. The Journal of international medical research, 2019. 47(7): p. 2961-2969	No	No follow-up time
213	Yenice S. Implementing a resource management program for accreditation process at the medical laboratory. Clinical Biochemistry, 2009. 42(4-5): p. 266-273.	No	No follow-up time
214	Yildiz O, Demirörs O. Measuring health care process quality with software quality measures. Studies in Health Technology and Informatics, 2012;180:1005–1009. PMID: 22874345.	No	No follow-up time
215	Ying Yuen V. Pediatric sedation: The Asian approach—current State of sedation in China, in Pediatric Sedation Outside of the Operating Room: A Multispecialty International Collaboration, Second Edition. 2015. p. 497-504.	No	Excluded after screening title
216	York D. Medical Tourism: The Trend toward Outsourcing Medical Procedures to Foreign Countries. Journal of Continuing Education in the Health Professions, 2008. 28(2): p. 99-102.	No	Excluded after screening title
217	Yousefian S. A Proposed adaptation of Joint commission international accreditation standards for hospital- JCI to the health care excellence model. Advances in Environmental Biology, 2013. 7(6): p. 956-967.	No	Excluded after screening abstract
218	Zubairi A., et. al. Workplace violence against doctors involved in clinical care at a tertiary care hospital in Pakistan. JPMA. The Journal of the Pakistan Medical Association, 2019. 69(9): p. 1355-1359.	No	Excluded after screening abstract

219	Astrup E. Passport to plastics: Cosmetic surgery tourism, medical malpractice, and the automatic establishment of personal jurisdiction by way of the joint commission international." Indiana Journal of Global Legal Studies 27(2): 347-369.	No	Excluded after screening title
220	Campra M, Riva P, Oricchio G, Brescia V. Association between patient outcomes and Joint Commission International (JCI) accreditation in Italy: An observational study. Calitatea - Access to Success, 2021;22(181):93–100	No	No follow-up time
221	De la Puente Pacheco MA. The Role of Outpatient Care Accreditation in Enhancing Foreign Patients' Perception of Colombian Medical Tourism: A Quasi-experimental Design." Inquiry (United States) 57.	No	Excluded after screening title
222	Gupta A. Evaluation of patients record and its implications in the management of trauma patients." Journal of Emergencies, Trauma and Shock 13(4): 257-263.	No	Excluded after screening title
223	Karakoc EY. Medical Tourism. GeoJournal Library. 121: 189-209.	No	Excluded after screening title
224	Kobayashi K., et al. Challenges for Joint Commission International accreditation: performance of orthopedic surgeons based on International Patient Safety Goals." Nagoya Journal of Medical Science 83(1): 87-92.	No	Excluded after screening title
225	Lee YH., et. al. Consensus survey on pre-procedural safety practices in radiological examinations: a multicenter study in seven Asian regions." The British journal of radiology 93(1113): 20200082.	No	Excluded after screening title
226	Poremski D., et. a. The Perspective of Key Stakeholders on the Impact of Reaccreditation in a Large National Mental Health Institute." Joint Commission Journal on Quality and Patient Safety 46(12): 699-705.	No	No JCI Accreditation
227	Shawan DA. The effectiveness of the joint commission international accreditation in improving quality at king fahd university hospital, saudi arabia: A mixed methods approach." Journal of Healthcare Leadership 13: 47-61.	Yes	
228	Steenbruggen R.A. Development of quality indicators for departments of hospital-based physiotherapy: a modified Delphi study." BMJ Open Quality 9(2).	No	Excluded after screening title
229	Westerway SC, Basseal JM. Endocavity Ultrasound Transducers: Why High-Level Disinfection Is Necessary. Ultraschall in der Medizin, 2022;43(2):204–208. DOI: 10.1055/a-1323-2345.	No	Excluded after screening title

230	De la Puente Pacheco A, Salazar Holguín N, Romero Barragán J. The Role of Outpatient Care Accreditation in Enhancing Foreign Patients' Perception of Colombian Medical Tourism: A Quasi-experimental Design. Inquiry, 2020; 57: 46958020964076. DOI: 10.1177/0046958020964076	No	Excluded after screening abstract
231	Cornistein, W. A., & Novau, G. (2004). Device-associated nosocomial infection rates in intensive care units in Argentina: Findings of the International Nosocomial Infection Control Consortium. Infection Control and Hospital Epidemiology, 25(3), 251–255. https://doi.org/10.1086/502386	No	Excluded after screening title
232	Pallares, C., et. al. Impact of Antimicrobial Stewardship Programs on Antibiotic Consumption and Antimicrobial Resistance in Four Colombian Healthcare Institutions. BMC Infectious Diseases, 22, 420. https://doi.org/10.1186/s12879-022-07410-6:contentReference[oaicite:7]{index=7}	No	Excluded after screening title
233	Aslanyan, L. A., & Truzyan, N. T. (2020). Challenges in Meeting Patient Assessment International Standards in Ambulatory Tuberculosis Services. The European Journal of Public Health, 30(Supplement_5), ckaa166.820.	No	Excluded after screening title
234	Akhmadyar, N. S., Khairulin, B. E., Amangeldy-Kyzy, S., & Ospanov, M. A. (2015). Developing drug formularies for the "National Medical Holding" JSC. Studies in Health Technology and Informatics, 210, 317–321. https://doi.org/10.3233/JRS-150686	No	Excluded after screening title
235	Al-Hashar A., et. al. Whose Responsibility is Medication Reconciliation: Physicians, Pharmacists or Nurses? A Survey in an Academic Tertiary Care Hospital. Saudi Pharmaceutical Journal. 2015;23(5):437–443. doi:10.1016/j.jsps.2015.06.012.	No	Excluded after screening title
236	Almidani E, Almohaisen K, Almaghrabi R, Alotaibi A, Almalki A. Improving medication reconciliation compliance at admission: A single department's experience. International Journal of Pediatrics and Adolescent Medicine, 2015;2(3):109–113. doi: 10.1016/j.ijpam.2015.10.001	No	Excluded after screening title
237	Batista, S. A., Miclos, P. V., Amendola, F., Bernardes, A., & Mohallem, A. G. C. (2021). Authentic Leadership, Nurse Satisfaction at Work and Hospital Accreditation: Study in a Private Hospital Network. Revista Brasileira de Enfermagem, 74(2), e20200227.	No	Excluded after screening title

238	Spear, J. M., Navarro, V. B., & Gayton, L. (2021). The Compliance Conversation: Navigating Variations in Sterile Processing Practices. AORN Journal, 114(5), 427–435. https://doi.org/10.1002/aorn.13533	No	Excluded after screening title
239	Evrenol Öçal, S., & Terzioğlu, F. (2022). Determining the Relationship Between Magnet Properties of Hospitals and the Professional Values of the Nurses: A Cross-sectional Study. Journal of Nursing Management, 30(4), 1027–1041.	No	Excluded after screening abstract
240	Rich, A., & Anderson, B. (2021). Educational training and nursing professional self-perception in Mongolia. Journal of Clinical Nursing, 30(21–22), 3210–3218. https://doi.org/10.1111/jocn.17211	No	Excluded after screening abstract
241	Yorke, A., Hunthausen, N., Paly, J. J., Carter, R. D., Yang, F., & Jhingran, A. (2021). Needs and Strengths Assessment for Radiotherapy Centers in Africa Transitioning to IMRT. International Journal of Radiation Oncology, Biology, Physics, 111(3), e350–e351.	No	Excluded after screening title
242	El Hadidi S., Hamdi M., Sabry N. Should pharmacists lead medication reconciliation in critical care? A one-stem interventional study in an Egyptian intensive care unit. Journal of Patient Safety. 2022;18(5):e895–e899. https://doi.org/10.1097/PTS.0000000000000983	No	Excluded after screening title
243	Tarier RRA, Zayyat R, Naoufal RN, Samaha HR. A case study exploring the impact of JCI standards implementation on staff productivity and motivation at the laboratory and blood bank. Health Science Reports. 2022;5(1):e497. https://doi.org/10.1002/hsr2.497	Yes	
244	El Khatib M, Hamidi S, Al Ameeri I, Al Zaabi H, Al Marqab R. Digital Disruption and Big Data in Healthcare – Opportunities and Challenges. ClinicoEconomics and Outcomes Research. 2022;14:563–574. https://doi.org/10.2147/CEOR.S369553	No	Excluded after screening title
245	Dempsey A., Robinson C., Moffatt N., Hennessy T., Bradshaw A., Teeling S. P., Ward M., McNamara M. Lean Six Sigma Redesign of a Process for Healthcare Mandatory Education in Basic Life Support—A Pilot Study. International Journal of Environmental Research and Public Health. 2021;18(21):11653. https://doi.org/10.3390/ijerph182111653	No	Excluded after screening abstract

246	Yang L., Xun Q., Xu J., et al. Application of the defect management improvement mode under Joint Commission International standard to improve the instrument cleaning and disinfection effect and management quality in the central sterile supply department: a randomized trial. Annals of Translational Medicine. 2022;10(2):137. https://doi.org/10.21037/atm-21-6772	No	No JCI Accreditation
247	Brouwers J., Seys D., Claessens F., Van Wilder A., Bruyneel L., De Ridder D., Eeckloo K., Vanhaecht K., Kesteloot K. The cost of a first and second hospital-wide accreditation in Flanders, Belgium. International Journal for Quality in Health Care. 2022;34(3):mzac062. https://doi.org/10.1093/intqhc/mzac062	No	No JCI Accreditation
248	Irfan M., Al Hashmi M., Al Nuaimi M., Al Zaabi H., Al Marqab R. Health research-strengthening and capacity development: Research support system model in an academic healthcare system. Journal of Multidisciplinary Healthcare. 2022;15:2063–2073. https://doi.org/10.2147/JMDH.S371222	No	Excluded after screening title
249	Al Mansour K. Hospital accreditation processes in Saudi Arabia: a thematic analysis of hospital staff experiences. BMJ Open Quality, 2021. 10(1): p. e001095. DOI: 10.1136/bmjoq-2020-001095. PMID: 33602834; PMCID: PMC7886531.	No	Excluded after screening abstract
250	Navarro-Escudero M., Organization and Implementation of a Stroke Center in Panamá: A Model for Implementation of Stroke Centers in Low and Middle Income Countries. Frontiers in Neurology, 2020. 11: p. 471. DOI: 10.3389/fneur.2020.00471. PMID: 32477283; PMCID: PMC7241571.	No	Excluded after screening title
251	Catalano A. Organizational Design: The Case of SARS-CoV-2 Vaccination in a Healthcare Centre. Sustainability, 2022; 14(3): 1381. DOI: 10.3390/su14031381.	No	Excluded after screening title
252	Kähkönen O., Nevalainen M., Airaksinen M., Juppo A. A pilot study about methods to reduce prescription errors in a chemotherapy day unit – Aspects to consider in pharmacist verification process. Exploratory Research in Clinical and Social Pharmacy, 2021; 3: 100057. DOI: 10.1016/j.rcsop.2021.100057.	No	Excluded after screening abstract
253	Paulino E.I., Santos L., Giordani F., et al. Costs and root causes of medication errors and falls in a teaching hospital: cross-sectional study. Revista Brasileira de Enfermagem, 2020; 73(Suppl 6): e20190432. DOI: 10.1590/0034-7167-2019-0432.	No	Excluded after screening abstract

254	Huapaya-Hueertas J., Atalaya-Quispe M., Ventura-Ortiz M. Effectiveness of the critical results application to support the diagnosis and therapy of chronic patients treated in a private clinic between 2017 and 2018. Archivos Peruanos de Cardiología y Cirugía Cardiovascular, 2020; 1(1): p. 13–18. DOI: 10.47658/arcacardiov.1i1.4.	No	Excluded after screening abstract
255	Anonymous. Erratum: Conflict of Interest Compliance Article 6. Global Journal on Quality and Safety in Healthcare, 2021; 4(3): p. 90. DOI: 10.4103/JQSH.JQSH_6_21.	No	Excluded after screening title
256	Sutinee. An Exploratory Study of Identifying Critical Success Factors for Customer Preferences in Medical Tourism Supply Chain using MAXQDA 2022. Proceedings of the International Conference on Business and Industrial Research (ICBIR), 2022; p. 340–345. DOI:	No	Excluded after screening title
257	Nal S. & Mustafa M. Mediating Role of Burnout in the Effect of Nurses' Workload on Turnover Intention during COVID-19 Pandemic. Journal of Nursing Management, 2021; 29(6): p. 1906–1913. DOI: 10.1111/jonm.13283.	No	Excluded after screening title
258	Hakim A. Proposal for a Normal Pressure Hydrocephalus Syndrome Center of Excellence. World Neurosurgery, 2020; 139: p. 276–280. DOI: 10.1016/j.wneu.2020.03.022.	No	Excluded after screening title
259	Sedova N. Russian healthcare in the development of medical tourism. Meditsinskoe Pravo, 2021; 3: p. 45–48.	No	Excluded after screening title
260	Darmer L. The effect of a VIPS implementation programme. Journal of Clinical Nursing, 2006; 15(3): p. 292–302.	No	No JCI Accreditation
261	Sicechi S. & Yand M. Evaluating Guidelines for Test Adaptation: A Methodological Approach. Procedia - Social and Behavioral Sciences, 2013; 78: p. 350–354.	No	Excluded after screening title
262	Tomson T. Valproate in the treatment of epilepsy in girls and women of childbearing potential. Epilepsia, 2019; 60(3): p. 479–482.	No	Excluded after screening title
263	Anonymous. The Impact of Outliers on Available Resources in a Teaching Hospital in Colombia. Journal of Healthcare Management, 2021; 66(Suppl 1): p. 44–50.	No	Excluded after screening title
264	Asmoro, Hariyati. Researching nurses' adherence to patient safety guidelines in emergency departments. International Journal of Nursing Practice, 2022; 28(1): e12957.	No	Excluded after screening abstract
265	Cece, M., & Kose, U. Comparative Analysis of Joint Commission International and Healthcare Information and Management Systems – Electronic Medical Record Adoption Model Measurement Models using Text Mining. Procedia Computer Science, 2021; 194: 499–508.	No	Excluded after screening title

266	Nusair A. Postmortem sampling time effect on toxicity biomarkers in rats exposed to an acute lethal methomyl dose. Toxicol Rep, 2021; 8: 1545–1552.	No	Excluded after screening title
267	Anonymous. Proceedings of Technology and Policy for Supporting Implementation of COVID-19 Response and Recovery Plan in Southeast Asia, ITTP-COVID19 2021. ITTP Conf Proc, 2021.	No	Excluded after screening title
268	Alanazi, A., & Milton, C. Exploring the rate and reasons for same-day cancellation of cardiac surgery after implementing Joint Commission International standards: a retrospective cross-sectional study. ITTP Conf Proc, 2021.	No	Excluded after screening title
269	Bao, Y., et al. Experience of the patient regarding their safety in the hospital environment. ITTP Conf Proc, 2021.	No	Excluded after screening abstract
270	Collado-Gonzales, M., Ferrero Garcia-Loygorri, J. Evolution of the perception of the safety culture of healthcare professionals in a pediatric emergency department. ITTP Conf Proc, 2021.	No	No JCI Accreditation
271	Conoscenti, E. Risk factors for surgical site infection following cardiac surgery in a region endemic for multidrug resistant organisms. ITTP Conf Proc, 2021.	No	Excluded after screening abstract
272	El Karaaoui, A. Using the SLIPTA checklist to assess laboratory readiness for Joint Commission International accreditation. ITTP Conf Proc, 2021.	No	Excluded after screening abstract
273	Alsamani, S. Challenges experienced during pharmacy automation and robotics implementation in JCI accredited hospital in the Arabian Gulf area: FMEA analysis–qualitative approach. ITTP Conf Proc, 2021.	No	Excluded after screening abstract
274	El Lithy, S. A. Benchmarking of medication incidents reporting and medication error rates in a JCI accredited university teaching hospital at a GCC country. ITTP Conf Proc, 2021.	No	Excluded after screening title
275	Gharleghi, B. & Supriyanto, E. Real-Time Transesophageal Echocardiography (TEE) Patient Safety Indicator Visualization According to Joint Commission International Standards. ITTP Conf Proc, 2021.	No	Excluded after screening title
276	Giri, S. & Poojary Coto, M. Preventing Risks of Infections and Medication Errors in IV Therapy (PRIME): A Patient Safety Initiative. ITTP Conf Proc, 2021.	No	Excluded after screening abstract
277	Hanymanthayya, M. Nursing Practice Improvement Strategies for Reducing Medication Errors. ITTP Conf Proc, 2021.	Yes	
278	Pallares, C. & Porras, A. Antimicrobial stewardship programs in seven Latin American countries: facing the challenges. ITTP Conf Proc, 2021.	No	Excluded after screening title

279	Radhakrishnan, R. Assessment on infection prevention and control knowledge among medical professionals in south Indian population. ITTP Conf Proc, 2021.	No	Excluded after screening title
280	Vuohijoki, A., et al. The Effects of Quality Assurance System Implementation on Work Well-Being and Patient Safety: Protocol for a Mixed Methods Study. ITTP Conf Proc, 2021.	No	Excluded after screening title
281	Zhang, H. Perceptions of Chinese Hospital Leaders on Joint Commission International Accreditation: A Qualitative Study. ITTP Conf Proc, 2021.	No	Excluded after screening abstract
282	Q. Safe medication management and use of narcotics in a Joint Commission International-accredited academic medical center hospital in the People's Republic of China. Ther Clin Risk Manag. 2016 Apr 6;12:535-44. doi:	No	Excluded after screening title
283	Brubakk K., et. al. A systematic review of hospital accreditation: the challenges of measuring complex intervention effects	No	Excluded after screening title
284	Valliani, K., et al. (2022). Determinant of repeat revascularization within 5 years of Percutaneous Coronary Intervention at a tertiary care hospital, Karachi: A matched case-control study. *Ann Med Surg (Lond)* 75: 103364.	No	Excluded after screening title
285	Vallone, D., et al. (2019). Incidence study of surgical site infections in a large University Hospital in Rome, first results. European Journal of Public Health 29.	No	Excluded after screening abstract
286	Virginio, L. and J. C. Dos Reis (2019). Finding Relations Between Requirements for Healthcare Information Systems Use in Hospitals: A Study on EMRAM and JCI.	No	Excluded after screening abstract
287	de Grande, R. S. and G. H. S. de Mendes (2015). Impacts of Joint Commission International Hospital Accreditation in a Brazilian hospital. Espacios 36(20): 10.	No	Excluded after screening abstract
288	jimeno, E., et al. (2018). Joint Commission International and implementation in CPR learning. Resuscitation 130: e70-e70.	No	Excluded after screening abstract
289	Anonymous: Health City Cayman Island. *Consulting - Specifying Engineer.*	No	Excluded after screening abstract
290	Shaw C, Groene O, Mora N, Sunol R. Accreditation and ISO certification: do they explain differences in quality management in European hospitals? Int J Qual Health Care. 2010 Dec;22(6):445-51. doi: 10.1093/intqhc/mzq054. Epub 2010 Oct 8. PMID: 20935006.	No	Excluded after screening abstract
291	Rathert C, May DR. Health care work environments, employee satisfaction, and patient safety: Care provider perspectives. Health Care Manage Rev. 2007 Jan-Mar;32(1):2-11. doi: 10.1097/00004010-200701000-00002. PMID: 17245197.	No	Excluded after screening abstract
