# Supplementary material for: Impact of joint commission international accreditation on occupational health and patient safety: A systematic review
Source: PLoS One. 2025 Jun 17;20(6):e0325894. doi: 10.1371/journal.pone.0325894 (PMC12173381; doi:10.1371/journal.pone.0325894)
Supplement: S5 File — (PDF) [file pone.0325894.s005.pdf]

First author	Intervention independent of other changes						Other risks	Total eval.
	D1	D2	D3	D4	D5	D6	D7	D8
Devkaran (2014)	Low	low	low	low	concerns	low	low	low
Devkaran (2015)	Low	low	low	low	concerns	low	low	Low
Devkaran (2019)	Low	low	low	low	concerns	low	low	Low
Fanelli (2017)	Concerns	high	concerns	no info	concerns	low	high	High
Halasa (2015)	Low	low	low	low	concerns	low	high	High
Inomata (2018)	No info	high	low	no info	concerns	concerns	high	high
Kaegan (2016)	No info	high	low	no info	concerns	concerns	no info	high
Kaegan (2014)	No info	concerns	low	no info	concerns	concerns	no info	high
Mekory (2017)	No info	low	low	no info	concernc	low	study design	high
Okumura (2019)	No info	concerns	low	no info	concerns	low	study design	high
Wang (2015)	No info	concerns	low	no info	low	low	concerns	concerns
Al Shawan (2021)	Low	low	low	no info	low	low	concerns	Low
Fang (2016)	No info	concerns	low	no info	low	low	concerns	concerns
Tarieh (2021)	Concerns	high	concerns	no info	no info	concerns	concerns	high
M Hanymanthayya (2023)	Concerns	concerns	low	concerns	low	low	high	high
