# Supplementary material for: Impact of joint commission international accreditation on occupational health and patient safety: A systematic review
Source: PLoS One. 2025 Jun 17;20(6):e0325894. doi: 10.1371/journal.pone.0325894 (PMC12173381; doi:10.1371/journal.pone.0325894)
Supplement: S6 File — (PDF) [file pone.0325894.s006.pdf]

CD, cannot determine; NA, not applicable; NR, not reported yes/ no	1. Was the study question or objective clearly stated?		2. Were eligibility/selection criteria for the study population prespecified and clearly described?		3. Were the participants in the study representative of those who would be eligible for the test/service/intervention in the general or clinical population of interest?		4. Were all eligible participants that met the prespecified entry criteria enrolled?		5. Was the sample size sufficiently large to provide confidence in the findings?		6.Was the test/service/intervention clearly described and delivered consistently across the study population?	
**Article**	**#1**	**#2**	**#1**	**#2**	**#1**	**#2**	**#1**	**#2**	**#1**	**#2**	**#1**	**#2**
Devkaran 2014	no	no	yes	yes	yes	yes	yes	yes	yes	yes	no	yes
Devkaran 2015	no	yes	yes	yes	yes	yes	yes	yes	yes	yes	no	yes
Devkaran 2019	no	yes	yes	yes	yes	yes	yes	yes	yes	yes	no	yes
Fanelli 2017	no	yes	no	yes	CD	no	CD	no	yes	yes	no	no
Halasa 2015	yes	yes	no	yes	yes	NR	CD	yes	no	yes	CD	no
Inomata 2018	yes	yes	yes	yes	no	yes	CD	yes	yes	yes	yes	yes
Kaegan 2016	yes	yes	yes	yes	no	no	yes	no	yes	no	yes	yes
Kaegan 2014	yes	yes	yes	yes	yes	yes	yes	no	yes	no	yes	yes
Mekory 2017	yes	yes	yes	yes	CD	yes	CD	yes	yes	NA	yes	yes
Okumura 2019	no	yes	no	yes	CD	yes	CD	yes	CD	yes	no	yes
Song 2014	yes	yes	no	yes	CD	CD	CD	yes	yes	yes	yes	yes
Wang 2015	yes	yes	yes	NR	CD	no	CD	yes	yes	yes	yes	yes
Al Shawan 2021	yes	yes	yes	yes	CD	yes	CD	no	yes	yes	yes	yes
Fang 2016	yes	yes	yes	no	CD	yes	CD	yes	yes	yes	yes	no
Tarieh 2021	yes	yes	CD	no	yes	yes	no	cd	no	no	yes	yes
Hanymanthayya 2023	yes	yes	no	no	no	yes	no	yes	NR	NR	no	no

7. Were the outcome measures prespecified, clearly defined, valid, reliable, and assessed consistently across all study participants?		8.Were the people assessing the outcomes blinded to the participants' exposures/interventions?		9. Was the loss to follow-up after baseline 20% or less? Were those lost to follow-up accounted for in the analysis?		10. Did the statistical methods examine changes in outcome measures from before to after the intervention? Were statistical tests done that provided p values for the pre-to-post changes?		11. Were outcome measures of interest taken multiple times before the intervention and multiple times after the intervention (i.e., did they use an interrupted time-series design)?		12. If the intervention was conducted at a group level (e.g., a whole hospital, a community, etc.) did the statistical analysis take into account the use of individual-level data to determine		Based on these assessments, the overall risk of the article – (Good, fair, poor quality)"		
**#1**	**#2**	**#1**	**#2**	**#1**	**#2**	**#1**	**#2**	**#1**	**#2**	**#1**	**#2**	**#1**	**#2**	**Final**
no	yes	CD	yes	CD	CD	yes	yes	yes	yes	yes	yes	fair/poor	good	good
no	yes	CD	yes	CD	CD	yes	yes	yes	yes	yes	yes	fair/poor	good	good
no	yes	CD	yes	CD	CD	yes	yes	yes	yes	yes	yes	fair	good	good
no	yes	CD	no	CD	no	no	NA	yes	no	CD	yes	poor	fair	poor
yes	yes	CD	yes	CD	NA	yes	yes	no	yes	CD	yes	fair	fair	fair
no	yes	CD	NR	CD	yes	yes	yes	no	yes	CD	yes	good	good	good
yes	yes	no	yes	yes	no	yes	yes	no	no	yes	NA	fair	fair	fair
yes	yes	no	NR	yes	no	yes	yes	no	no	yes	NA	good	fair	good
yes	yes	CD	no	CD	NA	yes	yes	no	yes	CD	yes	good	fair	good
no	yes	no	yes	CD	yes	yes	yes	no	yes	CD	yes	fair	good	good
yes	yes	NR	NR	CD	CD	yes	yes	yes	yes	yes	yes	good	good	good
yes	yes	CD	yes	CD	NR	yes	yes	no	yes	CD	NA	good	fair	good
yes	yes	CD	yes	CD	no	yes	yes	no	yes	CD	yes	good	good	good
yes	yes	CD	NR	CD	NR	yes	no	no	yes	CD	yes	good	fair/good	good
CD	CD	CD	CD	NR	NR	NR	NR	NR	NR	NR	NR	fair	fair/poor	poor
yes	yes	NA	NA	NR	NR	yes	yes	yes	yes	NR	CD	fair	fair	fair
